# Possible involvement of the oxLDL/LOX-1 system in the pathogenesis and progression of human intervertebral disc degeneration or herniation

**DOI:** 10.1038/s41598-017-07780-x

**Published:** 2017-08-07

**Authors:** Xinhua Li, Xuejun Wang, Zhouyang Hu, Zhaoxiong Chen, Haoxi Li, Xiaoming Liu, Zhi Yao Yong, Shanjing Wang, Zhanying Wei, Yingchao Han, Jun Tan, Cong Li, Xiao bo He, Guixin Sun, Desheng Wu, Lijun Li

**Affiliations:** 1 Department of Spinal Surgery, Shanghai East Hospital, Tongji University School of Medicine, 150 JiMo Road, Shanghai, 200120 China; 2 Department of Cardiovascular Medicine, Shanghai East Hospital, Tongji University School of Medicine, Shanghai, 200120 China; 3 Metabolic Bone Disease and Genetic Research Unit, Division of Osteoporosis and Bone Disease, Department of Endocrinology and Metabolism, Shanghai Jiao Tong University Affiliated Sixth People’s Hospital, Shanghai, China; 4 Department of medical imaging, Shanghai East Hospital, Tongji University, School of Medicine, 150 JiMo Road, Shanghai, 200120 China; 5 Department of Traumatology, Shanghai East Hospital, Tongji University, School of Medicine, 150 JiMo Road, Shanghai, 200120 China

## Abstract

Epidemiological studies have concluded that hyperlipidemia and atherosclerosis were related to intervertebral disc degeneration (IVDD). The presence of oxidized low density lipoprotein (ox-LDL) and the expression of lectin-like oxidized low density lipoprotein receptor 1 (LOX-1) have not been explored in this tissue. In this study, we investigated the presence of ox-LDL and the expression of its receptor LOX-1 in non-degenerated, degenerated or herniated human intervertebral discs (IVDs). The expression of LOX-1 and matrix metalloproteinase 3 (MMP3) were studied after incubating nucleus pulposus cells (NPCs) with ox-LDL. The presence of ox-LDL and LOX-1 was positively related with the extent of IVDD in nucleus pulposus (NP), end-plate cartilage and outer annulus fibrous, but not with the extent of degeneration of inter annulus fibrous. Ox-LDL significantly reduced the viability of human NPCs in a dose and time-dependent manner, and increased the expression of MMP3 induced by LOX-1. Pretreatment with anti-human LOX-1 monoclonal antibody reversed these effects. Ox-LDL, principally mediated by LOX-1, enhanced MMP3 production in NPCs through the NF-κB signaling pathway. In conclusion, increased accumulation of ox-LDL and LOX-1 in IVDs indicates a specific role of the receptor-ligand interaction in degeneration or herniation of IVDs.

## Introduction

Back pain is a leading cause of disability and job-related disability^1, 2^. Lumbar disc herniation (LDH) is a major cause of low back pain and sciatica^3^. Though the etiology and treatment of intervertebral disc degeneration (IVDD) has been extensively investigated, the underlying pathophysiologic mechanism remains unclear^4–7^. Abnormal lipid metabolism and atherosclerosis (AS) were implicated to be critical players in the development of age-related degenerative diseases^8, 9^.

IVDD is a common age-related disease. As in other age-related degenerative diseases, serum lipid levels and AS were positively correlated with LDH. There are two main hypotheses on how abnormal lipid metabolism and AS can cause LDH. Firstly, dyslipidemia can accelerate the AS process and its morbidity, which will destroy vascular supply to the already poorly vascularized human intervertebral discs (IVDs). Secondly, release of inflammatory cytokines caused by dyslipidemia and AS may be another potential pathogenetic mechanism. To some extent, the IVDD was regarded as one inflammatory-related joint disease. However, the precise pathophysiological mechanism underlying these associations remains unclear^10–14^.

Oxidized low density lipoprotein (Ox-LDL) accumulation under oxidative stress conditions plays a very important role in the development of AS^15^. Ox-LDL has many biological functions; it causes lipid accumulation, elicits pro-inflammatory responses, promotes apoptosis, and increases protease activity^16^. Lectin-like oxidized low density lipoprotein receptor 1 (LOX-1) is a type II membrane protein that belongs to the C-type lectin family, and can act as a cell-surface receptor for ox-LDL^17^. LOX-1 is expressed in various cells, including endothelial cells, macrophages and chondrocytes, and its expression is enhanced by proinflammatory cytokines such as interleukin-1 (IL-1). Previous studies suggested that ox-LDL can decrease cell viability^18^, induce reactive oxygen species production^19^, reduce proteoglycan synthesis^20^, increase matrix metalloproteinase 3 (MMP3) production^21^ and monocyte chemoattractant protein-1 (MCP-1) expression^22^ in human or bovine chondrocytes through LOX-1. Supporting the possible involvement of lipid peroxidation in the pathogenesis of IVDD, the lipid peroxidation inhibitors such as vitamin C inhibited degradation of the extracellular matrix (ECM) in nucleus pulposus cell (NPC) monolayer cultures [3]. However, the specific mechanism remains largely unknown. Previous studies showed that vitamin C could prevent ox-LDL binding to LOX-1 in osteoarthritis (OA). However, whether vitamin C has similar effects on NPCs remains largely unknown.

Current evidence implicates major pathological changes in a degenerating disc to begin with proteoglycan breakdown, cell loss and diminished water-binding capacity of the nucleus pulposus (NP)^23^. The breakdown of proteoglycan can be due to the decreased ability of NPCs to synthesize ECM and increased activity of matrix metalloproteinases (MMPs). MMPs are critical enzymes involved in the destruction of ECM of IVDs. Among the MMPs, MMP3 is regarded as a critical enzyme, which can degrade proteoglycan and fibronectin, and activate proMMPs^24^. Although the correlation between ox-LDL, LOX-1 and MMP3 is implicated in rheumatoid arthritis (RA) and OA^21, 25^, its role in the pathophysiology of IVDD remains unknown.

In this study, we investigated whether ox-LDL/LOX-1 ligand-receptor system was involved in IVD degeneration or herniation, and the effects of ox-LDL on cell viability and MMP3 production in cultured human NPCs.

## Results

### Patients demographics

Twenty-four disc samples were obtained by lumbar surgery (lumbar fracture or lumbar disc herniation). Patients demographics (age, serum ox-LDL and LDL) in the study and control groups are shown in Table 1. The average age of the patients was 45.08 ± 15.76 years, range 25–73 years. The average serum ox-LDL was 480.43 ± 279.98 mU/ml for non-degenerated IVDs (histological degeneration scores 0 to 3), 728.67 ± 256.69 mU/ml for intermediate-degenerated IVDs (histological degenerative scores 4 to 8), and 705.69 ± 185.43 mU/ml for severely degenerated IVDs (histological degenerative scores 9 to 12). The average serum LDL was 3.24 ± 1.32 mmol/L for non-degenerated IVDs, 3.21 ± 0.49 mmol/L for intermediate-degenerated IVDs, and 3.19 ± 0.67 mmol/L for severely degenerated IVDs. Therefore, the serum ox-LDL and LDL levels were not positively correlated with the extent of degenerated IVDs.

**Table 1:** Patient details and grades of tissues used for immunohistochemistry analysis.

Subject	Age	Vertebral level	Histological grade	Clinical diagnosis	serum LDL (mmol/L)	serum oxLDL (mU/ml)
1	28	L1-L2	1	lumbar fracture	1.65	405.90
2	37	L2-L3	1	lumbar fracture	2.88	266.10
3	29	L3-L4	2	lumbar fracture	4.13	664.67
4	30	L3-S4	2	lumbar fracture	3.83	867.83
5	26	L2-S3	2	lumbar fracture	1.93	97.74
6	25	L4-L5	3	disc herniation	5.02	580.34
7	39	L3-L4	4	disc herniation	3.22	989.31
8	35	L3-L4	4	disc herniation	3.51	804.80
9	27	L5-S1	5	disc herniation	2.94	170.40
10	31	L5-S1	5	disc herniation	3.84	488.35
11	36	L4-L5	6	disc herniation	3.50	580.34
12	28	L4-L5	6	disc herniation	3.20	804.80
13	47	L4-L5	7	disc herniation	2.80	989.31
14	52	L4-L5	7	disc herniation	3.23	937.99
15	54	L5-S1	8	disc herniation	3.76	825.08
16	55	L5-S1	8	disc herniation	2.15	696.39
17	56	L3-L4	9	disc herniation	2.95	713.02
18	59	L3-L4	9	disc herniation	2.87	555.38
19	55	L5-S1	10	disc herniation	3.08	804.80
20	59	L4-L5	10	disc herniation	4.13	664.67
21	65	L4-L5	10	disc herniation	3.75	555.38
22	67	L4-L5	11	disc herniation	3.10	890.37
23	69	L5-S1	11	disc herniation	3.70	397.85
24	73	L5-S1	11	disc herniation	1.97	913.74

### Immunohistochemical localization

**Figure 1:**
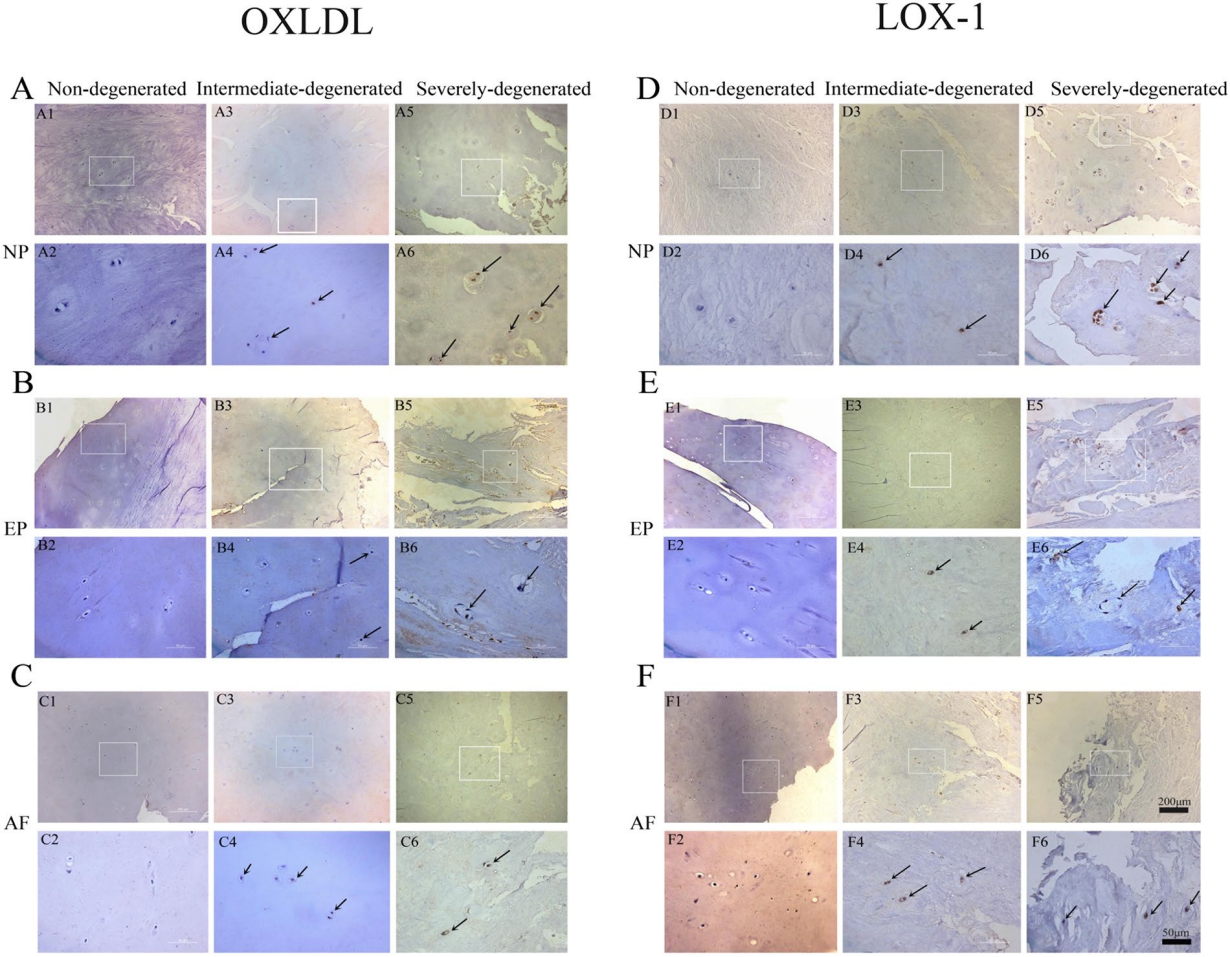
Examples of immunohistochemical staining for LOX-1 (1:100, Abcam, USA) and oxLDL (1:100, biorbyt, USA) in human intervertebral disc. LOX-1 (row **A**,**B**,**C**) and oxLDL (row **D**,**E**,**F**) in non-degenerate discs (A1,A2,B1,B2,C1,C2,D1,D2,E1,E2,F1,F2); intermediate degenerated disc: (A3,A4,B3,B4,C3,C4,D3,D4,E3,E4,F3,F4); Severe degenerated disc: (A5,A6,B5,B6,C5,C6,D5,D6,E5,E6,F5,F6). Immunopositivity is revealed by brown staining. In non-degenerate discs, no cell clusters were seen and little immunopositivity was observed in the single cells. In degenerated discs, a large number of cell clusters were observed, which were predominately immunopositive. Bars = 200 μm (row 1,3,5), Bars = 50 μm (row 2,4,6). A one-way ANOVA were used for statistical assessments. (*P < 0.05, **P < 0.01).

The LOX-1 and ox-LDL immunopositive cells were found in normal and degenerated discs, but the proportion was significantly higher in the degenerated discs (Fig. 1). When normal IVDs were examined, slight immunoreactive staining was found with anti-LOX-1 and anti-ox-LDL antibodies. Non-specific control rabbit IgG yielded no positive staining in the IVDs (data not shown).

### Immunohistochemical staining and quantification of immunopositive cells

**Figure 2:**
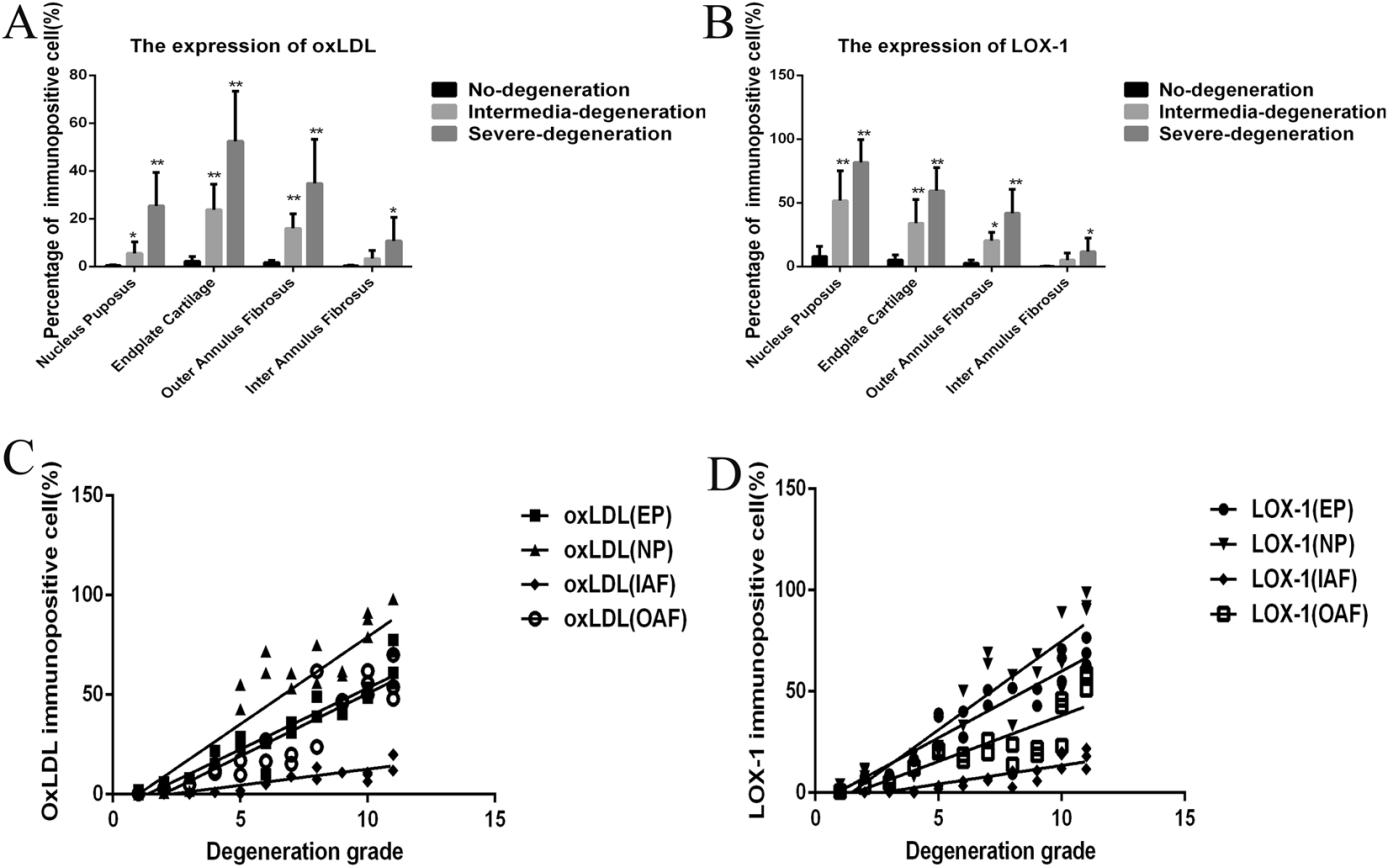
The percentage of cells with immunopositivity for (**A**) LOX-1, (**B**) oxLDL, according to location in the disc and grade of intervertebral disc degeneration. For oxLDL immunopositive cell. (**C**) The Correlations analysis between ox-LDL immunoreactivity and intervertebral disc degeneration score were performed.(R^2^_EP_ = 0.91, P < 0.0001; R^2^_NP_ = 0.83, P < 0.0001; R^2^_OAF_ = 0.85, P < 0.0001; R^2^_IAF_ = 0.75, P < 0.0001). (**D**) The Correlations analysis between LOX-1 and intervertebral disc degeneration score were performed (R^2^_EP_ = 0.83, P < 0.0001; R^2^_NP_ = 0.86, P < 0.0001; R^2^_OAF_ = 0.76, P < 0.0001; R^2^_IAF_ = 0.78, P < 0.0001). Data are presented as means ± SD. (*P < 0.05, **P < 0.01).

The most prominent aspects of the immunophenotype of non-degenerated IVDs (histological degeneration scores 0 to 3) was slight immunoreactivity for ox-LDL and LOX-1 (Fig. 2). In the intermediate-degenerated IVDs (histological degenerative scores 4 to 8), the immunophenotype of cells differed in two ways from cells in non-degenerated IVDs (scores 0 to 3). Firstly, the proportion of cells immunopositive for LOX-1 and ox-LDL were higher than that of cells from non-degenerated IVDs, and this immunopositivity increased with the severity of degeneration in NPC, endplate cartilage (EPC), and outer annulus fibrosus (OAF). Secondly, the LOX-1 and ox-LDL immunopositive cells in the internal annulus fibrosus (IAF) showed no significant difference between the non-degenerated IVDs and intermediate-degenerated IVDs (histological degenerative scores 4 to 8). In the severely degenerated IVDs (histological degenerative scores 9 to 12), the proportion of cells immunopositive for LOX-1 and ox-LDL were significantly higher than that of non-degenerated IVDs.

Notably, the mean percentage of ox-LDL-positive-immunostained NPCs was 0.41% in the non-degenerated IVDs, 2.23% in the intermediate degenerated IVDs, and 25.34% in the severely degenerated IVDs. The ox-LDL-positive-immunostaining for EPC was 2.23% in the non-degenerated IVDs, 23.8% in the intermediate degenerated IVDs, and 52.41% in the severely degenerated IVDs. For OAF, these percentages were 1.7%, 15.98% and 34.77%; while for IAF, these percentages changed to 0.3%, 3.29% and 10.7%.

The mean percentage of LOX-1-positive-immunostained NPCs was 7.98% in the non-degenerated IVDs, 51.73% in the intermediate degenerated IVDs and 81.64% in the severely degenerated IVDs. The LOX-1-positive-immunostaining for EPC was 5.2% in the non-degenerated IVDs, 33.97% in the intermediate degenerated IVDs and 59.42% in the severely degenerated IVDs. For OAF, these percentages were 2.73%, 20.28% and 41.77%; while for IAF, these percentages changed to 0.17%, 5.08% and 11.7%.

The correlation analysis between ox-LDL immunoreactivity and IVD degeneration score was performed (R^2^_EP_ = 0.91, P < 0.0001; R^2^_NP_ = 0.83, p < 0.0001; R^2^_OAF_ = 0.85, p < 0.0001; R^2^_IAF_ = 0.75, p < 0.0001). The correlation analysis between LOX-1 and IVD degeneration score was performed (R^2^_EP_ = 0.83, p < 0.0001; R^2^_NP_ = 0.86, p < 0.0001; R^2^_OAF_ = 0.76, p < 0.0001; R^2^_IAF_ = 0.78, p < 0.0001). Data are presented as means ± SD (*p < 0.05, **p < 0.01).

### Effect of ox-LDL on cell viability

**Figure 3:**
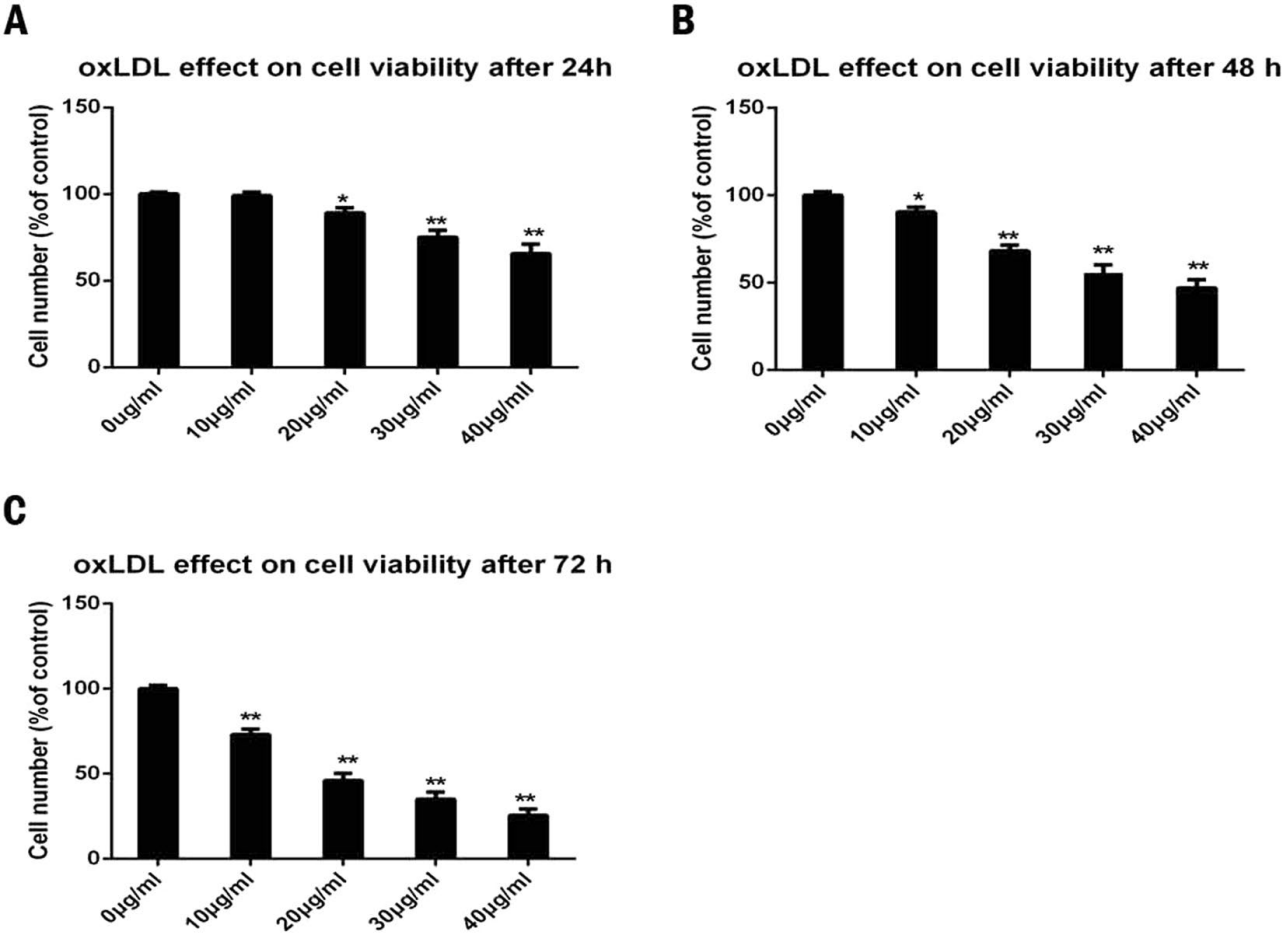
OxLDL induced apoptotic cell death in nucleus pulposus with CCK8 assay. (**A**,**B**,**C**) Effect of increasing concentrations of ox-LDL (0–40 μg/ml) on nucleus pulposus cells viability after 24 h (**A**), 48 h (**B**) and 72 h (**C**). The cells without ox-LDL treatment were served as control. The cell number were decreased into 99.1% for 10 μg/ml, 89.1% for 20 μg/ml, 75.12% for 30 μg/ml, 65.6% for 40 μg/ml after incubating with oxLDL for 24 h. The cell number were decreased into 90.39% for 10 μg/ml, 68.17% for 20 μg/ml, 55.28% for 30 μg/ml, 47.12% for 40 μg/ml after incubating with oxLDL for 48 h and into 73.1% for 10 μg/ml, 46.1% for 20 μg/ml, 35.12% for 30 μg/ml, 25.6% for 40 μg/ml after incubating with oxLDL for 72 h. Cell numbers are expressed as a percentage of the controls. Data are shown as the mean ± SD of three independent experiments. A one-way ANOVA were used for statistical assessments. (*P < 0.05, **P < 0.01).

We investigated the effect of ox-LDL on the viability of NPCs (The result in relation to the identification of NPCs could be found in the supplement files). Increasing concentrations of ox-LDL (10 μg/ml, 20 μg/ml, 30 μg/ml and 40 μg/ml) were added to human NPCs and cell viability was assessed by the CCK8 assay after 24 h, 48 h and 72 h (p < 0.05). ox-LDL dramatically reduced the viability of NPCs in a time and dose-dependent manner (Fig. 3). The cell number decreased to 99.1% for 10 μg/ml, 89.1% for 20 μg/ml, 75.12% for 30 μg/ml and 65.6% for 40 μg/ml after incubating with ox-LDL for 24 h. The cell number decreased to 90.39% for 10 μg/ml, 68.17% for 20 μg/ml, 55.28% for 30 μg/ml and 47.12% for 40 μg/ml after incubating with ox-LDL for 48 h, and into 73.1% for 10 μg/ml, 46.1% for 20 μg/ml, 35.12% for 30 μg/ml and 25.6% for 40 μg/ml after incubating with ox-LDL for 72 h. We repeated the experiments with cells from three patients and obtained similar results.

### Enhanced MMP3 expression induced by LOX-1 in ox-LDL-stimulated NPCs

**Figure 4:**
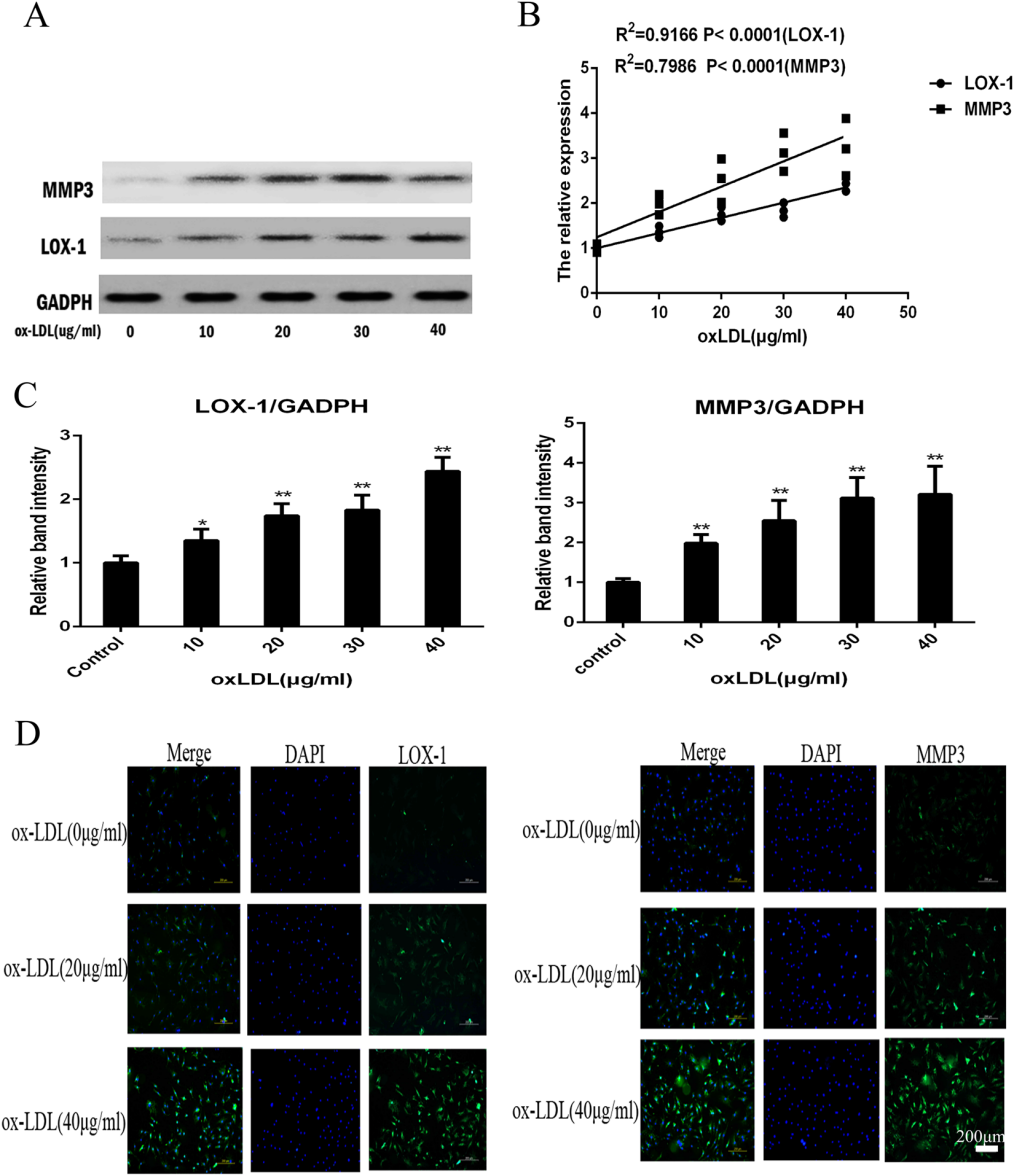
OxLDL can increase the expression of LOX-1 and MMP3. (**A**,**C**) Western blotting analysis for the protein expressions of LOX-1 and MMP3. Nucleus pulposus cells were treated with 48 h for different concentration periods (0–40 μg/ml). The LOX-1 and MMP3 was quantified. Data were presented as mean ± SD from three independent experiments. The cells without oxLDL treatment were served as control. (**B**) The Correlations analysis between LOX-1 and intervertebral disc degeneration score were performed (R^2^_LOX-1_ = 0.92, P < 0.0001; R^2^_MMP3_ = 0.80, P < 0.0001). Data are presented as means ± SD. (**D**) Representative fluorescent microscopic images of Hoechst/PI double stained nucleus pulposus cells showing that the increasing concentration of oxLDL (only show 0 μg/ml, 20 μg/ml and 40 μg/ml) can promote the expression of LOX-1 and MMP3. Bars = 200μm. (*P < 0.05, **P < 0.01).

We used ox-LDL (10 μg/ml, 20 μg/ml, 30 μg/ml and 40 μg/ml) to stimulate NPCs for 48 h, and the levels of MMP3 and LOX-1 were determined by western blotting and immunofluorescence. The expression of LOX-1 and MMP3 increased in NPCs in a dose-dependent manner as detected by western blotting and immunofluorescence (p < 0.05). Increased LOX-1 and MMP3 expression was evident after stimulation with 10 μg/ml ox-LDL and persisted at 40 μg/ml (Fig. 4).

**Figure 5:**
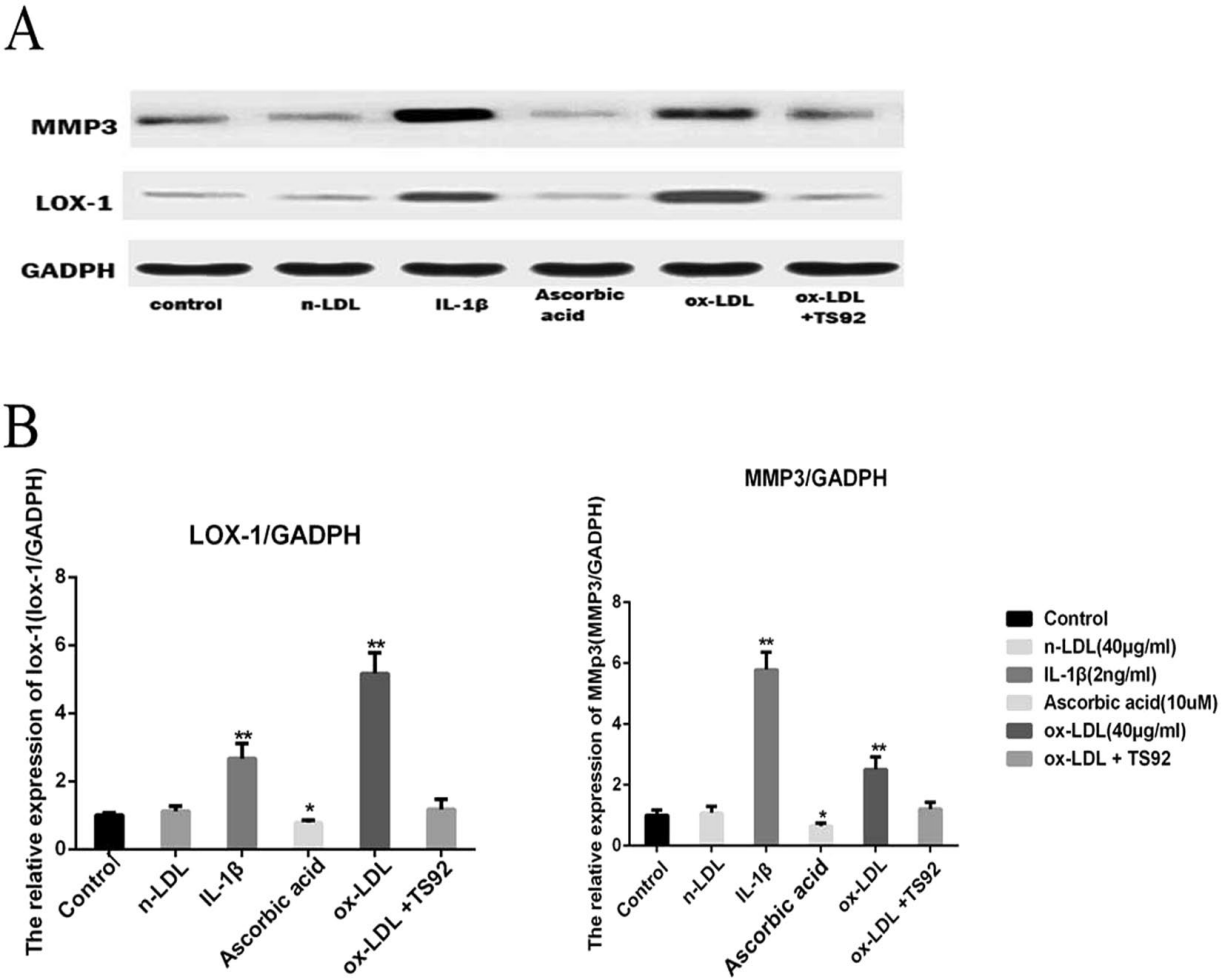
(**A**) Western blotting analysis for the protein expressions of LOX-1 and MMP3. NPC were respectively incubated with 40 μg/ml n-LDL, 2ng/ml IL-1β, 10 μM ascorbic acid, 40 mg/ml ox-LDL, or preincubated with 15 mg/ml anti-human LOX-1 mAb (TS92) for 24 h than stimulated with ox-LDL for 48 h. (**B**) The expression of LOX-1 and MMP3 was detected by western blotting. Data were presented as mean ± SD from three independent experiments. The cells without oxLDL treatment were served as control. A one-way ANOVA were used for statistical assessments. (*P < 0.05, **P < 0.01).

To examine whether LOX-1-induced MMP3 expression was stimulated by ox-LDL in NPCs, the expression of MMP3 and LOX-1 in NPCs was investigated by western blotting after stimulation with 40 μg/ml n-LDL, 2 ng/ml IL-1β (Proteintech, USA), 10 uM ascorbic acid (Sigma, USA), 40 μg/ml ox-LDL and pretreatment with 15 μg/ml anti-human LOX-1 monoclonal antibody (TS92) for 24 h followed by stimulation with 40 μg/ml ox-LDL for 48 h. As shown in Fig. 5, IL-1β and ox-LDL induced the expression of LOX-1 and MMP3 (p < 0.05); n-LDL did not affect LOX-1 and MMP3 expression (p > 0.05); LOX-1 and MMP3 expression decreased after addition of ascorbic acid (p < 0.05); and pretreatment of NPCs with TS92 significantly suppressed the increase in expression of LOX-1 and MMP3 induced by ox-LDL.

### Ox-LDL increased the expression of MMP3 by activating the NF-κB signaling pathway

NPCs were treated with 40 μg/ml ox-LDL for different time periods (0, 15, 30 and 45 min). The expression levels of NF-κB-p65 (P65) and phospho-NF-κB-p65 (PP65) were quantified. The expression of PP65/actin increased in NPCs in a time-dependent manner as detected by western blotting. The correlation analyses between the stimulation duration and the expression of P65, PP65 were performed (R^2^_PP65_ = 0.00128, p < 0.9121; R^2^_P65_ = 0.952, p < 0.0001).

**Figure 6:**
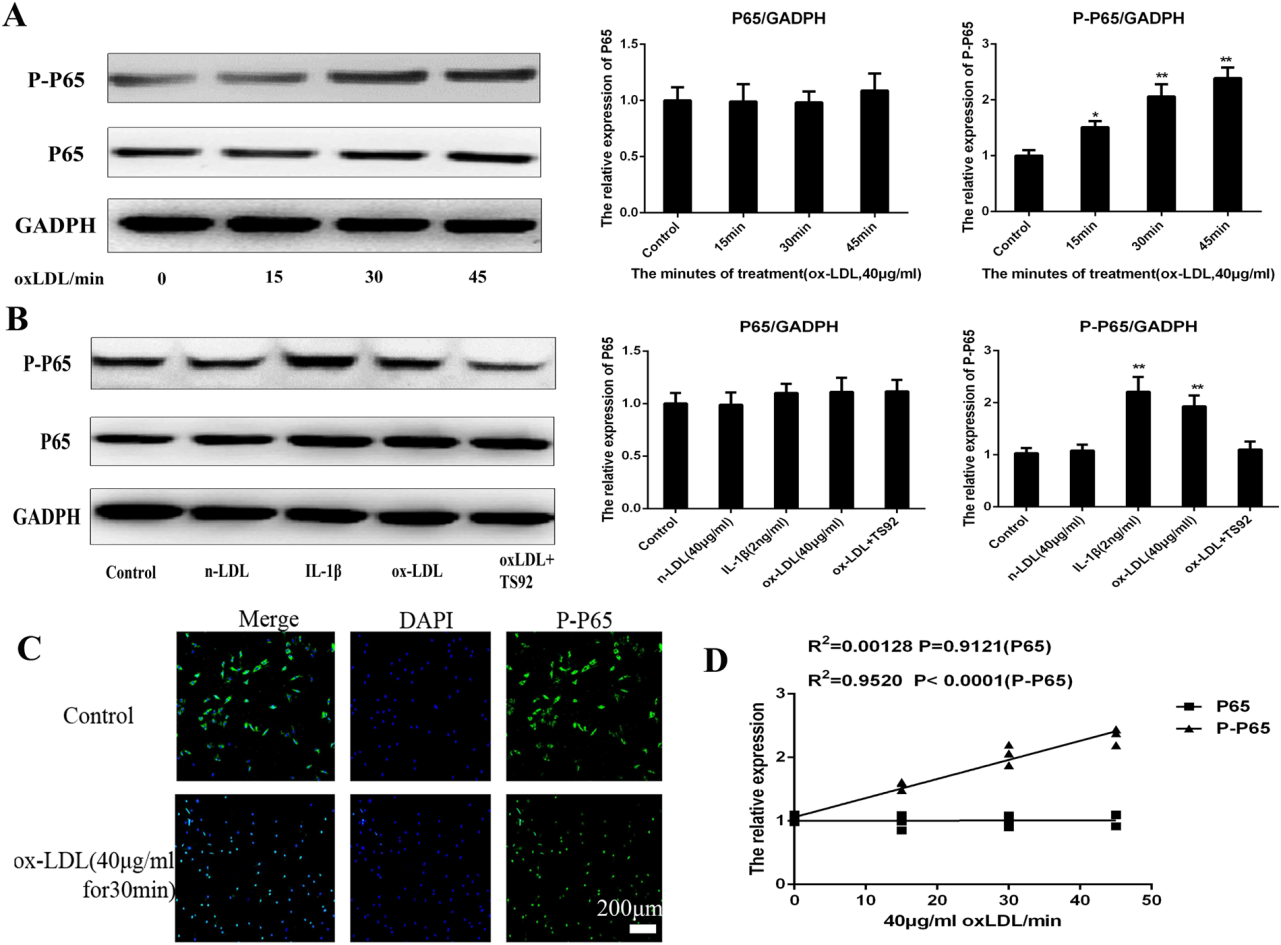
OxLDL can increase the expression of MMP3 by activating the NF-κB signal pathway. (**A**) Western blotting analysis for the protein expressions of P65 and PP65. Nucleus pulposus cells were treated with 40 μg/ml oxLDL for different periods (0, 15, 30, 45 min). The expressions of P65 and PP65 was quantified. Data were presented as mean ± SD from three independent experiments. (**B**) Western blotting analysis for the protein expressions of P65 and PP65. NPC were respectively incubated with 40 μg/ml n-LDL, 2 ng/ml IL-1β, 10 μM ascorbic acid, 40 mg/ml ox-LDL, or preincubated with 15 mg/ml anti-human LOX-1 mAb (TS92) for 24 h than stimulated with ox-LDL for 48 h. The cells without oxLDL treatment were served as control. It shows that IL-1β and ox-LDL could improve the expression of PP65 (P < 0.01); n-LDL did not affect PP65 expression (P > 0.05) and pretreatment of NPCs with TS92 significantly suppressed the increase in expression of PP65 by ox-LDL. A one-way ANOVA were used for statistical assessments. (**C**) Representative fluorescent microscopic images of Hoechst/PI double stained nucleus pulposus cells showing that the 40 μg/ml oxLDL can promote the PP65 enter into the cell nuclear from cytoplasm from 0–45 min (only show 30 min). Bars = 200 μm. (**D**) The Correlations analysis between the stimulate period and the expression of P65, PP65 were performed (R^2^PP65 = 0.00128, P < 0.9121; R^2^P65 = 0.952, P < 0.0001). Data are presented as means ± SD. (*P < 0.05, **P < 0.01).

NPCs were incubated with 40 μg/ml n-LDL, 2 ng/ml IL-1β, 10 μM ascorbic acid, 40 mg/ml ox-LDL, or preincubated with 15 mg/ml anti-human LOX-1 mAb (TS92) for 24 h and then stimulated with ox-LDL for 48 h. The cells without ox-LDL treatment served as controls. As shown in Fig. 6, IL-1β and ox-LDL induced the expression of PP65 (p < 0.01); n-LDL did not affect PP65 expression (p > 0.05) and pretreatment with TS92 significantly suppressed the increase in expression of PP65 induced by ox-LDL.

Immunofluorescence of Hoechst/PI double-stained NPCs showed that 40 μg/ml ox-LDL could promote PP65 entry into the nucleus from the cytoplasm in 0–45 min.

## Discussion

The progression of IVDs by serum lipids and atherosclerosis is thought to be associated with the decrease in nutrition caused by the narrowing of arteries, which indirectly leads to IVDD^14, 26^. The IVD is located at the end of the nutrient chain, which makes it the first structure to suffer from insufficient nutrient supply^26–29^. However, whether lipids directly affect the structural destabilization of IVD matrix remains unclear. Among the abnormally metabolized lipids, ox-LDL is considered to be the most harmful, since its oxidative modification represents the initial event in atherogenesis^15, 30^.

Previous studies suggested that ox-LDL/LOX-1 system played an important role in the development of RA and OA^18–22, 31–35^. Knocking down the LOX-1 gene relieved the progression of OA in mice^36^. In our study, the mean percentages of LOX-1 and ox-LDL immunopositive cells in IVD samples increased with the IVDD grades, as measured by the degree of morphological degeneration. These results suggested that the ox-LDL/LOX-1 ligand-receptor system may be involved in the pathogenesis and progression of IVDD. Interestingly, we found that the number of LOX-1 and ox-LDL immunopositive cells in IAF showed no significant difference between the non-degenerated and intermediate-degenerated IVDs, which could be due to the small sample size.

To investigate the relationship between circulating LDL, ox-LDL and the presence of ox-LDL in IVDs with the state of degeneration, the serum LDL and ox-LDL levels in the study participants were measured. The serum LDL and ox-LDL was not associated with the state of degeneration and the presence of ox-LDL in IVDs. The small sample size may contribute to these vague results. In fact, ox-LDL is usually found in the blood as lipoproteins or bound to carrier plasma proteins in order to increase their solubility. In this way, lipids become part of a large complex, which restricts their transport through cartilage since size is a determining factor for molecule diffusion^30^. The large complex of ox-LDL may access the IVDD by the following three ways. Firstly, it is likely that ox-LDL in the systemic circulating blood^37^ enters the IVDs through the EP and OAF vasculature. Furthermore, with the degeneration of IVDs, numerous large molecules such as hyaluronan and ox-LDL can penetrate the cartilage matrix after IL-1 and other inflammatory mediator treatment^38^. Secondly, LDL in the systemic circulating blood might enter and get oxidized in the inflamed IVD^20^. The resultant ox-LDL might then penetrate the IVDs matrix and cells. Thirdly, LDL penetrating the degraded cartilage matrix might be oxidatively modified by ROS produced by IVD cells^39, 40^ and the resultant ox-LDL could associate with LOX-1 in IVD cells.

Interestingly, we found that LOX-1-positive IVD cells were more widely distributed than ox-LDL-positive cells, which indicates that LOX-1 may be induced by other ligands such as proinflammatory cytokines (TNFα, IL-1β, IFN-γ and CRP), modified lipoproteins (ox-LDL and LPC), high glucose, advanced glycation end-products, growth factors, mechanical stimuli such as cyclic tensile stress, etc^15, 18, 31^. In the present study, IL-1β and ox-LDL could upregulate the expression of LOX-1. Other related factors that induce the expression of LOX-1 in IVDs are being currently investigated.

Having established a basis for a functional excess of ox-LDL/LOX-1 ligand-receptor system in degenerated and herniated IVDs, we then investigated the role of ox-LDL/LOX-1 ligand-receptor system in the processes that characterize disc degeneration, namely, increased apoptosis and production of MMPs.

We observed that 10–40 μg/ml ox-LDL can reduce the viability of NPCs. Previous studies have shown that ox-LDL induces apoptosis of endothelial cells through the NF-κB pathway, and non-apoptotic cell death in association with Akt activation in articular chondrocytes of rats^41^. In addition, ox-LDL and cyclic tensile stretch load can induce LOX-1 expression in human chondrocytes, resulting in decreased cell viability and proteoglycan synthesis^18, 20, 31^.

MMP3 has been suggested to play a very important role in IVDD. Studies on RA and endothelial cells have demonstrated that ox-LDL can enhance the expression of MMP3^21, 42^. A recent study conducted in IVDs of an ApoE knockout mice model also suggested that hyperlipidemia can increase the expression of MMP3 and accelerate IVDD^43^. This is the first study to show that ox-LDL can enhance the expression of MMP3 in human NPCs through LOX-1 in a dose-dependent manner. To further confirm the inter-relationship between MMP3, ox-LDL, and LOX-1, we incubated NPCs with IL-1β, nLDL, anti-human LOX-1 monoclonal antibody TS92, and ascorbic acid.

The addition of vitamin C to NPCs resulted in a decrease in expression of LOX-1 suggesting that this anti-oxidative nutrient has the ability to stop the cycle of ox-LDL binding to LOX-1. Vitamin C has been shown to play a role in ECM production^44^, as it is required for the synthesis of type II collagen, the most abundant protein in IVD, which moderately stimulates the synthesis of aggrecan. Its absence has been associated with reduced mechanical resilience of collagen fibrils and increased turnover rates^45^. Although vitamin C is known to facilitate repair of IVDD, the specific mechanism remains unclear. Suppressing the production of LOX-1 and MMP3 induced by ox-LDL may be an important mechanism.

In this study, IL-1β and ox-LDL could improve the expression of PP65, an important transcription factor in the NF-κB signaling pathway (p < 0.01). Meanwhile, immunofluorescence study showed that ox-LDL can promote PP65 entry into the nucleus from cytoplasm in 0–45 min. These results suggested that NF-κB signaling pathway plays a critical role in the expression of MMP3 induced by ox-LDL in NPCs. The effect of pretreatment with anti-human LOX-1 monoclonal antibody TS92 on NPCs also confirmed this result. NF-κB controls the expression of numerous proinflammatory molecules, including cytokines tumor necrosis factor-α (TNF-α), interleukin-1β (IL-1β), interleukin-6 (IL-6), chemokines (interleukin-8, IL-8), and adhesion molecules (intercellular adhesion molecule-1 and vascular cell adhesion molecule-1)^19^. In chronic cartilage-related degenerative diseases such as OA, the negative regulatory loop with its inhibitor, IkB-α is overwhelmed by the positive loop involving NF-κB activation by TNF-α, IL-1β, and NF-κB-dependent expression of these two major proinflammatory cytokines. Indeed, IVDD is associated with persistent *in situ* NF-κB activity^46^. In the context of chronic cartilage-related degenerative disease, induction of NF-κB activity in NPCs would facilitate degradation of the ECM of IVDs. In conclusion, ox-LDL binding to LOX-1 in human NPCs activated NF-κB and increased MMP3 expression.

These observations support the hypothesis that hypercholesterolemia is a risk factor of arthritis, and lipid peroxidation products such as ox-LDL are involved in cartilage matrix degradation in OA and IVDD.

However, there were some limitations in this study. Firstly, the sample size was relatively small. Secondly, some tissues were collected from herniated discs. The changes and cellular processes associated with degeneration in early stages or within the intact disc are somewhat distinct from those in herniated discs because the herniated disc tissues have been exposed to numerous factors outside the IVD environment (i.e., vascularity, inflammation) for variable periods of time.

## Conclusion

This is the first study to show that colocalization of IVDs is associated with the presence of ox-LDL and LOX-1 expression. In addition, ox-LDL can up-regulate MMP3 production induced by LOX-1 in human NPCs. The *in vivo* and *in vitro* studies indicated that the ox-LDL/LOX-1 ligand-receptor system may be involved in the pathogenesis and progression of IVD degeneration or herniation. LOX-1 could be a promising target in the treatment of IVD degeneration or herniation.

## Methods

### Tissue samples

Specimens were derived from surgical disc procedures performed on patients with fresh herniated discs, degenerative disc disease, and lumbar burst fracture. **All the methods were performed in accordance with relevant guidelines and regulations. All the participants consented to participate in this study**. All IVDs consisted of full-thickness wedges of 120° of anteriorly removed arc. Surgical specimens were immediately transported to the laboratory in a sterile tissue culture medium (for cell cultures) or placed in 10% neutral buffered formalin (for histological studies).

### Primary cultures of human nucleus pulposus cells

The human NPCs were dissected from surgical disc procedures performed on individuals with lumbar burst fracture (without degenerated disc, patient No. 1, 3 and 4 in Table 1). NPCs were isolated and cultured as previously described^47^. After isolation, NPCs were re-suspended in Dulbecco’s modified Eagle’s medium/F-12 (HyClone, USA) containing 10% fetal bovine serum (Gibco, USA) and 50 units/ml of penicillin and streptomycin (HyClone, USA), and then incubated at 37 °C in a humidified atmosphere with 95% air and 5% CO_2_. The confluent cells were detached by trypsinization, seeded into 25 cm^2^ cell culture box in complete culture medium (DMEM supplemented with 10% FBS, 100 mg/ml streptomycin and 50 U/ml penicillin) in a 37 °C and 5% CO_2_ environment. The medium was changed every three days. At 90% confluency, the second passage human NPCs were cultured under serum-free conditions for 24 h for subsequent experiments.

### Treatment of tissue specimens

Specimens were fixed in 10% neutral-buffered formalin for 24 hours. As some specimens contained bone, all the samples were decalcified in ethylenediaminetetraacetic acid (EDTA) (Beyotime) for 14 days^48^. The specimens were embedded in paraffin. The slides were deparaffinized in xylene and hydrated through graded alcohols to distilled water. Slices from the tissue blocks were subjected to H&E staining to score the degree of morphological degeneration according to previously published criteria^49^. Based on this scoring, discs were selected to represent a range of scores from non-degenerated (0 to 3) to the most severe level of degeneration (12). A score of 0–3 represents a histologically normal disc, 4–8 indicates intermediate degeneration and 9–12 suggests severe degeneration.

### Detection of ox-LDL and LOX-1 proteins by immunohistochemistry

In the quantitative determination of serum ox-LDL and LDL levels, the samples were analyzed using a specific enzyme-linked immunosorbent assay (Huamei, Wuhan, China) according to the manufacturer’s instructions. Ox-LDL and LDL concentrations were determined in duplicate.

Immunohistochemistry (IHC) was performed as previously described^50^. Endogenous peroxidase was blocked using 3% H_2_O_2_ in methanol. Slides were incubated in 10% serum (of the species in which the secondary antibody was generated) and then incubated with anti human ox-LDL (1:100, Biorbyt, USA) and anti human LOX-1 (1:100, Abcam, USA) at 4 °C overnight. Negative controls were treated with normal rabbit IgG (Sigma, USA) under the same conditions. After washing, the slides were incubated with the secondary antibody for 40 minutes at room temperature followed by addition of avidin–biotin–peroxidase complex. The samples were visualized with 0.025% diaminobenzidine solution and counterstained with Mayer’s hematoxylin solution.

### Image analysis

All slides were visualized using a Leica (Leica, Cambridge, UK) RMDB research microscope. The images were captured using a digital camera and analyzed using the Bioquant Nova image analysis system (Bioquant, Nashville, TN, USA). Within each area, 200 cells were counted and the number of immunopositive cells (brown-stained cells), expressed as a proportion of total cells, were calculated for disc sections grouped with the scores 0–3, 4–8 and 9–12. Data was presented on graphs as means ± SD.

### Preparation of native LDL (nLDL) and ox-LDL

Human nLDL was isolated from freshly donated plasma by sequential ultracentrifugation, as previously described^17^.

### Preparation of ox-LDL

Native LDL (200 μg/ml) was oxidized by exposure to CuSO_4_ (5 μmol/l free Cu^2+^) in phosphate-buffered saline at 37 °C for 20 h. Oxidation was terminated by refrigeration. The oxidative state was confirmed by measuring the quantity of thiobarbituric acid-reactive substances (TBARS) (nmol/mg protein). Agarose gel electrophoresis showed increased electrophoretic mobility and minimal aggregation of ox-LDL particles. The fluorescent carbocyanine dye DiI was conjugated with ox-LDL according to the manufacturer’s instructions^17^.

### Cell counting kit assay (CCK-8)

Human NPCs were seeded in 96-well plates and incubated in the presence of increasing concentrations of ox-LDL (10–40 μg/ml) for 24 h, 48 h, and 72 h. Cell survival rate was determined using the established cell counting kit (CCK-8) assay. Each well was incubated with the CCK-8 solution (Sigma) for 4 h, and the absorbance was measured at 590 nm using a spectrophotometer. The number of viable cells per well in the presence of ox-LDL was compared with untreated control cells. Wells containing only medium served as blank controls.

### Western Blot

The expression levels of proteins were determined by western blot analysis of total protein extracts from NPCs. Cell samples were lysed in RIPA buffer, sonicated, and protein concentrations were calculated using the BCA protein assay kit. Proteins were loaded onto 8% SDS-PAGE gels and transferred to PVDF membranes (Millipore, Billerica, MA, USA). After blocking for 1 hour, the membranes were incubated with primary antibodies overnight at 4 °C. Primary antibodies specific to LOX-1 (1:1000, Abcam), MMP3 (1:1000, Cell Signaling Technology) and β-actin (1:1000, Cell Signaling Technology) were used. Negative controls were performed with normal rabbit IgG (Sigma) under the same conditions. After washing with Tris Buffered Saline with Tween (TBST) for three times, the membranes were incubated with the respective secondary antibodies. Then the bands were detected with ECL plus reagent (Millipore) by the ChemiDoc™ XRS + System (BIO-RAD, USA). Relative expression levels of proteins were determined by quantitative densitometric analysis using image analysis software (Image lab, Bio-Rad, USA).

### Immunofluorescence

The cells were fixed in 10% neutral-buffered formalin for 15 min. After perforation and sealing, the cells were incubated with anti-LOX-1 antibody (1:200, Abcam, USA) and anti-MMP3 antibody (1:200, Cell Signaling Technology), overnight at 4 °C, washed three times for 5 min each, and then incubated with secondary anti-rabbit antibody conjugated with Alexa Fluor 488 for 1 h at room temperature. Negative controls were treated with normal rabbit IgG (Sigma, USA) under the same conditions. Images were obtained with a Fluo view confocal microscope and prepared with Photoshop software.

### Statistical analysis

Results are presented as means ± SD. GraphPad Prism 6.02 software was used to perform the statistical analysis. The combined results of the individual experiments were used to prepare the Figures, and data were presented as mean ± 95% CI (lower limit, upper limit) for the three experiments. Analyses of variance, Scheffe’s test, and one-way ANOVA were used for statistical assessments. The correlation analyses were performed. p < 0.05 was considered to be statistically significant, p < 0.01 was considered to be extremely statistically significant.

### Ethical Approval

Experimental study of disc specimens were approved prospectively by the authors’ human subjects Institutional Review Board. (**Institutional Review Board of shanghai east hospital**).

**Informed consent:** All of the participants consented to participate in this study.

**Consent for publication**

Not applicable **Availability of supporting data**

## Electronic supplementary material

supplement
