# Supplementary material for: Possible involvement of the oxLDL/LOX-1 system in the pathogenesis and progression of human intervertebral disc degeneration or herniation
Source: Sci Rep. 2017 Aug 7;7:7403. doi: 10.1038/s41598-017-07780-x (PMC5547039; doi:10.1038/s41598-017-07780-x)
Supplement: Supplementary file 1 — supplement [file 41598_2017_7780_MOESM1_ESM.pdf]

Possible involvement of the oxLDL/LOX-1 system in pathogenesis and progression of human intervertebral disc degeneration or herniation

Xinhua Li[a] , Xuejun Wang[b], Zhouyang Hu[a] , Zhaoxiong Chen[a] , Haoxi Li[a],Xiaoming Liu[a], ZhiYao Yong[a] , Shanjing Wang[a], Zhanying Wei[c], Yingchao Han[a] , Jun Tan[a] , Cong Li [a], Xiao bo He[d], Guixin Sun[E]*, Desheng Wu[a]*, Lijun Li[a]*

a Department of Spinal Surgery, Shanghai East Hospital, Tongji University School of Medicine, 150 JiMo Road, Shanghai 200120, China.

b Department of Cardiovascular Medicine, Shanghai East Hospital, Tongji University School of Medicine, Shanghai, 200120, China.

c Metabolic Bone Disease and Genetic Research Unit, Division of Osteoporosis and Bone Disease, Department of Endocrinology and Metabolism, Shanghai Jiao Tong University Affiliated Sixth People's Hospital, Shanghai, China

d Department of medical imaging , Shanghai East Hospital East Hospital, Tongji University, School of Medicine, 150 JiMo Road, Shanghai 200120, China

e Department of Traumatology, Shanghai East Hospital, Tongji University, School of Medicine, 150 JiMo Road, Shanghai 200120, China

*Correspondence author:

Li jun Li : Department of Spinal Surgery, East Hospital, Tongji University , School of Medicine, 150 JiMo Road, Shanghai 200120, China.

Phone number: 021-38804518, Fax number: 021-58798999, E-mail: liliju@163.com

Desheng Wu: Department of Spinal Surgery, East Hospital, Tongji University , School of Medicine, 150 JiMo Road, Shanghai 200120, China.

Phone number: 021-38804518, Fax number: 021-58798999, E-mail: eastspinesci@163.com

Gui xin Sun: Department of Traumatology, East Hospital, Tongji University, School of Medicine, 150 JiMo Road, Shanghai 200120, China

Phone number: 021-38804518, Fax number: 021-58798999, E-mail:tjdxyxyjs@163.com

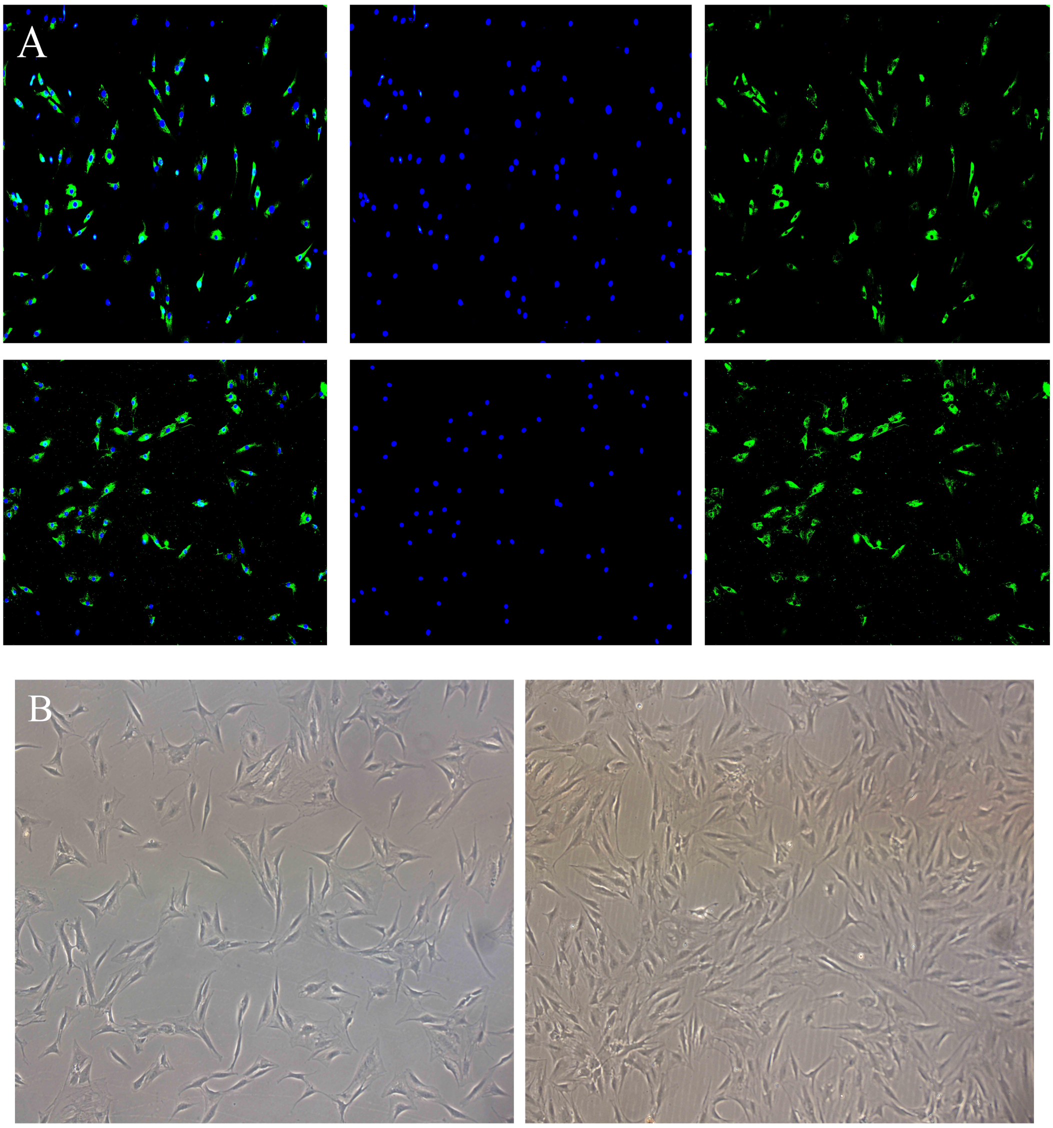

Supplement data: The identify of NPCs’ phenotype character by immunofluorescence and morphology. A:Representative fluorescent microscopic images of Hoechst/PI double stained nucleus pulposus cells with aggrecan. The cells appear aggrecan-positive staining were recognized as NPC. The The purity of NPC= aggrecan-positivecell/all cells(counted by DAPI)*100%. As result, the purity of NPC reached 98% after calculating1000 cells. B: The morphology of NPCs.
